# Human pleural fluid is a potent growth medium for *Streptococcus pneumoniae*

**DOI:** 10.1371/journal.pone.0188833

**Published:** 2017-11-30

**Authors:** Natalia D. Popowicz, Sally M. Lansley, Hui M. Cheah, Ian D. Kay, Christine F. Carson, Grant W. Waterer, James C. Paton, Jeremy S. Brown, Y. C. Gary Lee

**Affiliations:** 1 Pharmacy Department, Sir Charles Gairdner Hospital, Perth, Western Australia, Australia; 2 Division of Medicine, University of Western Australia, Perth, Western Australia, Australia; 3 Institute for Respiratory Health, University of Western Australia, Perth, Western Australia, Australia; 4 Department of Microbiology and Infectious Diseases, PathWest Laboratory Medicine, Royal Perth Hospital, Perth, Western Australia, Australia; 5 Respiratory Department, Royal Perth Hospital, Perth, Western Australia, Australia; 6 Research Centre for Infectious Diseases, Department of Molecular and Cellular Biology, University of Adelaide, Adelaide, South Australia, Australia; 7 Centre for Inflammation and Tissue Repair, Respiratory Medicine, University College London, London, England; 8 Respiratory Department, Sir Charles Gairdner Hospital, Perth, Western Australia, Australia; Instituto Butantan, BRAZIL

## Abstract

Empyema is defined by the presence of bacteria and/or pus in pleural effusions. However, the biology of bacteria within human pleural fluid has not been studied. *Streptococcus pneumoniae* is the most common cause of pediatric and frequent cause of adult empyema. We investigated whether *S*. *pneumoniae* can proliferate within human pleural fluid and if growth is affected by the cellular content of the fluid and/or characteristics of pneumococcal surface proteins. Invasive *S*. *pneumoniae* isolates (n = 24) and reference strain recovered from human blood or empyema were inoculated (1.5×10^6^CFU/mL) into sterile human malignant pleural fluid samples (n = 11). All *S*. *pneumoniae* (n = 25) strains proliferated rapidly, increasing by a median of 3009 (IQR 1063–9846) from baseline at 24hrs in all pleural effusions tested. Proliferation was greater than in commercial pneumococcal culture media and concentrations were maintained for 48hrs without autolysis. A similar magnitude of proliferation was observed in pleural fluid before and after removal of its cellular content, p = 0.728. *S*. *pneumoniae* (D39 strain) wild-type, and derivatives (n = 12), each with mutation(s) in a different gene required for full virulence were inoculated into human pleural fluid (n = 8). *S*. *pneumoniae with* pneumococcal surface antigen A *(ΔpsaA*) mutation failed to grow (2207-fold lower than wild-type), p<0.001, however growth was restored with manganese supplementation. Growth of other common respiratory pathogens (n = 14) across pleural fluid samples (n = 7) was variable and inconsistent, with some strains failing to grow. We establish for the first time that pleural fluid is a potent growth medium for *S*. *pneumoniae* and proliferation is dependent on the PsaA surface protein and manganese.

## Introduction

Pneumonia affects 450 million patients worldwide each year, [1] with *Streptococcus pneumoniae* the most common bacterial cause. As many as 20–40% of patients with pneumonia develop a simple parapneumonic pleural effusion [2]. Pleural infection can develop when the pleural fluid is secondarily infected, and affects ~80,000 people in the UK and USA a year with significant morbidity and mortality [3].

Empyema represents the most severe end of the pleural infection spectrum. It is defined by the presence of bacteria, or pus, in the pleural fluid. *S*. *pneumoniae* accounts for the vast majority of pediatric empyema and is among the most frequent causative organisms of empyema in adults [4–6]. The incidence of pneumococcal empyema continues to rise globally [7–9].

Compared to most other pathogens that cause pneumonia, *S*. *pneumoniae* seems to have a particular affinity for causing pleural infection, but the reasons why are not fully understood. During pleural infection, the pleural fluid is exudative and contains a diverse milieu of biologically active molecules. Despite the clinical importance of empyema, no studies have investigated the biological interactions between bacteria and pleural fluids. It is unknown, for example, if bacteria are merely shed from the pleural tissues into the fluid and if the fluid influences bacterial growth. On one hand, exudative fluids are rich in nutrients that can potentially enhance microbial growth; on the other hand, many proteins and enzymes (eg defensins, and lysozymes etc.) in the fluid can serve to defend against bacterial invasion. These questions are difficult to study in empyema fluid as the presence of bacteria can profoundly alter the pleural fluid composition. In clinical settings, patients are often given systemic antibiotics before the fluid is sampled, taking away the opportunity to study the effects of bacterial growth.

Pleural effusion is not unique to pleural infection but is the common end-product of a variety of other non-infective pleural diseases/inflammation that leads to vascular hyper-permeability and plasma leak into the pleural cavity. In this paper, we studied whether the pleural fluid of patients with other non-infective pleural etiologies could support survival and growth of *S*. *pneumoniae* strains. We hypothesized that pleural fluid can serve as a growth medium for common empyema bacteria such as *S*. *pneumoniae*. We further investigated the role of common pneumococcal surface proteins, in particular nutrient transporters, on pneumococcal growth and survival within human pleural fluid.

## Materials and methods

### Pleural fluid samples

Pleural fluid samples were collected from patients attending Sir Charles Gairdner Hospital (SCGH) that required pleural fluid drainage for clinical indications, with approval (SCGH Human Research Ethics Committee 2012–156).

Pleural fluid samples were collected using aseptic techniques from patients with pleural effusions of non-infective etiologies. All samples were subjected to routine bacterial culture for which the fluid was injected into and transported in blood culture bottles and processed in an accredited hospital laboratory (PathWest, Western Australia). Pleural samples confirmed to be culture-negative were used in experiments. Patients who received antibiotics within 72 hours were excluded. Pleural fluid biochemistry (pH, protein, lactate dehydrogenase and glucose) was measured as previously described [10]. Supernatant was obtained from pleural fluid by centrifugation at 1020×*g* for 10 minutes.

Pleural fluids (n = 53) were collected from 45 patients; effusions obtained from the same individual (n = 8) were collected on separate occasions at least 7 days apart. Most samples were malignant pleural effusions (n = 47, 88.7%) and 10.6% (n = 5) were transudates; (S1 Table). Samples were stored between 2–8°C and used within 96hrs of drainage.

### Bacterial strains and inoculums

Clinical isolates of *S*. *pneumoniae* (n = 24) from patients with invasive pneumococcal infection were obtained from PathWest (Royal Perth Hospital, WA). Serotypes of the isolates include type 1, 11A and 19F from pleural fluid and 1, 6B, 6C, 8 (n = 3), 10A, 11A, 12F, 19A (n = 8), 21, 22F (n = 2) and 35B from blood cultures. In addition, a *S*. *pneumoniae* reference strain of capsular serotype 3 (CIP 104225, ATCC^®^6303, American Type Culture Collection, Manassas, VA, USA) was used (Table 1).

**Table 1:** List of *Streptococcus pneumoniae* serotypes and other bacteria used in this study.

Source	Capsular Serotype	Bacteria	Source
*S*. *pneumoniae* clinical isolates		Reference strains	
Pleural fluid	1		
Pleural fluid	11A	*Streptococcus anginosus*	ATCC^®^ 10556^™^
Pleural fluid	19A	*Streptococcus anginosus*	ATCC^®^ 33397^™^
Blood culture	1	*Streptococcus intermedius*	ATCC^®^ 27335^™^
Blood culture	6B	*Enterococcus faecalis*	NCTC^®^ 8213^™^
Blood culture	6C	*Staphylococcus aureus*	ATCC^®^ 9144^™^
Blood culture	8 (n = 3)	*Escherichia coli*	ATCC^®^ 105365^™^
Blood culture	10A	*Escherichia coli*	ATCC^®^ 25922^™^
Blood culture	11A	*Moraxella catarrhalis*	NCTC^®^ 3622^™^
Blood culture	12F	*Pseudomonas aeruginosa*	ATCC^®^ 25668^™^
Blood culture	19A (n = 7)	Clinical isolates	
Blood culture	19F		
Blood culture	21	*Methicillin-resistant Staphylococcus aureus*	Clinical isolate
Blood culture	22F (n = 2)	*Pseudomonas aeruginosa*	Clinical isolate
Blood culture	35B	*Pseudomonas aeruginosa*	Clinical isolate
*S*. *pneumoniae* reference strain		*Klebsiella oxytoca*	Clinical isolate
ATCC^®^ 6303^™^	3	*Klebsiella pneumoniae*	Clinical isolate

ATCC, American Type Culture, Collection, Manassas, VA, US; NCTC, National Collection of Type Cultures, Salisbury UK.

Bacterial reference strains (n = 9) including *Streptococcus anginosis* ATCC^®^10556 and ATCC^®^33397, *S*. *intermedius* ATCC^®^27335, *Enterococcus faecalis* NCTC^®^8213 (National collection of type culture, Salisbury UK), *Staphylococcus aureus* ATCC^®^9144, *Escherichia coli* ATCC^®^105365 and ATCC^®^25922, *Moraxella catarrhalis* NCTC^®^3622, *Pseudomonas aeruginosa* ATCC^®^25668 and clinical isolates (n = 5) capable of causing invasive disease including methicillin-resistant *S*. *aureus*, *P*. *aeruginosa* (n = 2), *Klebsiella pneumoniae* and *Klebsiella oxytoca* (PathWest) were used for comparison growth studies with *S*. *pneumoniae* (Table 1).

Wild-type D39 *S*. *pneumoniae*, a capsule type-2 isolate, and its derivatives (n = 11) with mutations in genes required for full virulence have previously been described [11–20], and those used are listed in Table 2. Mutant strains were selected for using antibiotics (10μg/ml chloramphenicol, 0.2μg/mL erythromycin and/or 500μg/mL kanamycin) where necessary. Bacteria were stored in broth containing 10%(v/v) glycerol at -80°C.

**Table 2:** Characteristics of D39 *S*. *pneumoniae* mutants used across experiments.

D39 Mutant	Name	Virulence role	Construction	Reference
Δ*ply*	Pneumolysin	Host cell cytotoxin Diverts complement activity	In frame deletion mutant	[11]
Δ*lytA*	Autolysin	Major autolysin of S. *pneumoniae* Mediates the release of pneumolysin and possible inflammatory cell wall degradation products	Insertion-duplication mutation	[12]
Δ*pspA*	Pneumococcal surface protein A	Prevents complement mediated opsonisation Inhibits lactoferrin	Insertion-duplication mutation	[13]
Δ*cbpD*	Choline binding protein D	Competence induced cell lysis and therefore affects subsequent DNA release	Insertion-duplication *mutation*	[16]
Δ*luxS*	Biosynthesis of type 2 autoinducer AI-2	Quorum sensing Affects translocation of bacteria	Insertion-duplication mutation	[15]
Δ*psaA*	Pneumococcal surface antigen A	Manganese uptake Resistance to oxidative stress	Insertion-duplication mutation	[14]
Δ*pitA*	Putative iron uptake lipoprotein	Iron uptake	Insertion duplication mutation	[17]
Δ*piuB/piaA*	Iron Uptake ABC transporter	Iron uptake	Insertion duplication mutation	[18]
Δ*adcAI*	Zinc uptake ABC transporter	Zinc uptake	Deletion mutant	[19]
Δ*adcAII/adcA*	Zinc uptake ABC transporter	Zinc uptake	Deletion mutant	[19]
Δ*livH*	Branched-chain amino-acid (BCAA) transporter mutant	BCAA uptake	Deletion mutant	[20]

To standardize counts between experiments, frozen inocula of *S*. *pneumoniae* were prepared as previously described [21]. Inocula for D39 strains and non-pneumococcal bacteria were prepared from 18-hour blood agar to a turbidity of 0.5 MacFarland (PathWest) in sodium chloride 0.85% using a Sensititre Nephelometer (Thermo-Scientific; Waltham, USA).

### Pleural fluid inoculation

Pleural fluid samples (in 3mL aliquots) were warmed to 37°C then inoculated with approximately 1.5×10^6^ CFU/mL of bacteria and incubated for 24hrs at 37°C and 5% CO_2_ unless specified. Baseline inoculum and final concentrations (CFU/mL) were verified by manual counting log serial dilutions from blood agar.

Pleural fluid characteristics and bacteria used in each experiment are presented in Table 3. Pleural fluids before and after centrifugation (n = 11 pairs), to remove cells, were inoculated with *S*. *pneumoniae* isolates (n = 25). Six pairs were inoculated within 6 hours from collection and all within 24 hours (range 1 to 18 hours). Growth was compared in parallel to Todd-Hewitt Broth (THB; Thermo-Scientific) containing 17% FCS (Serana Australia, WA), [21], Dulbecco’s Modified Eagle Medium (DMEM; Invitrogen, NY) containing 1000mg/L of glucose and sodium chloride 0.85% (n = 3 each) over 48 hours in six pleural fluid samples at 4, 8, 12, 18, 24, 28 and 48 hours. A further 7 pleural fluids were inoculated with non-pneumococcal bacteria (n = 14) and *S*. *pneumoniae* clinical isolate (n = 2) controls, at the same starting inoculum of 1.5×10^6^ CFU/mL, for comparison purposes. The CFU/mL of bacteria failing to grow at 24 hours was subsequently measured at 4 and 8 hours in pleural fluid (n = 3).

**Table 3:** Pleural fluid characteristics for individual experiments.

*Experiment*	*S*. *pneumoniae* in pleural fluid	*S*. *pneumoniae* in different medium	Other bacterial pathogens	*S*. *pneumoniae* D39 mutants* including Δ*psaA*	*S*. *pneumoniae* D39 nutrient transporters**	*S*. *pneumoniae* Δ*psaA +* manganese (Mn)
**Pleural Fluid Samples**, n	**11**	**6**	**7**	**8**	**8**	**16**
**Pleural Fluid ID** †	1–11	12–17	47–53	18–25	26–33	31–46
**Number of bacterial strains used**	25 *S*. *pneumoniae*	3 *S*. *pneumoniae*	14 non-pneumococci 2 *S*. *pneumoniae*	1 D39 wild-type 6 D39 mutants*	1 D39 wild-type 5 D39 mutants**	1 D39 wild 1 D39 Δ*psaA*
**Number of aliquots inoculated**	275 pleural + cells 275 pleural—cells	18 pleural + cells 27 other media	112 pleural + cells	56 pleural + cells	48 pleural + cells	32 pleural + cells 32 pleural + cells +Mn
**Sex**, male (%) **Age**, mean ±SD	7 (63.6%) 63 years (SD±)]	3 (50%) 70 years (SD±14)	4 (57.1%) 74 years (SD±4)	3 (37.5%) 70 years (SD±10)	7 (87.5%) 69 years (SD±8)	13 (81.2%) 69 years (SD±8)
**pH**, mean (SD)	7.33 (SD±0.11)	7.28 (SD±0.11)	7.37 (SD±0.11)	7.34 (SD±0.15)	7.33 (SD±0.12)	7.32 (SD±0.12)
**LDH**, U/L median (IQR)	303 (116–4350)	251 (218–397)	245 (82.5–392)	319 (189–752)	391 (200–574)	379 (200–574)
**Protein**, g/L median (IQR)	39 (36–44)	31 (23–39)	31 (24–35)	34 (26–40)	39 (17–41)	39 (17–40)
**Glucose**, mmol/L, mean (SD)	4.09 (SD±1.9)	4.9 (SD±1.8)	6.3 (SD±2.3)	4.9 (SD±2.4)	8.4 (SD±8.4)	6.8 (SD±6.0)

^**†**^ Individual pleural fluid characteristics are presented in S1 Table.

*Δ*ply*, Δ*lytA*, Δ*pspA*, Δ*psaA*, Δ*luxS*, Δ*cbpD*,

** Δ*pitA*, *ΔpiuB/piaA*, *ΔadcAI*, *ΔadcAII/adcAI*, *ΔlivH*

D39 wild and mutant pneumococci (n = 12) were tested in pleural effusions (n = 8). Further experiments with *ΔpsaA* in parallel to wild type in pleural fluids (n = 16) with and without supplementation of 3μM manganese chloride tetrahydrate (Sigma-Aldrich, Sydney, Australia) were performed. The concentration was determined following concentration dependent supplementation experiments ranging from 0.003 to 30μM of manganese.

### Statistical analysis

Statistical analyses were performed using SigmaPlot 12.5 (Systat Software, San Jose, CA). Results are presented as mean (SD) or median (IQR) based on normality of data. The Wilcoxon Signed-Rank test and paired t-test (for non-parametric and parametric data respectively) were used to compare baseline to 24 hour pneumococcal concentrations and Student’s t-test to compare growth between pleural fluid and other medium at various time-points over 48 hours. The Mann-Whitney Rank Sum test was used to compare differences between samples containing cells and correspondent supernatant and the Pearson Correlation was used to determine any relationship between spun and unspun samples. Significance was defined as p<0.05.

## Results

### Optimization and pneumococcal growth conditions

Significant growth was observed when 3mL volumes of pleural fluid were inoculated with 1.5 × 10^6^ CFU/mL *S*. *pneumoniae* (n = 3), with a median increase of 6875 (IQR 3494–15744) fold at 24 hours compared to baseline. Volumes ranging from 1 to 10mL in both tubes and multi-well plates demonstrated similar growth, however volumes less than 1mL had inconsistent or poor growth (data not shown). Inoculum concentrations in the range of 1.5 x 10^3^ to 1.5x 10^6^ CFU/mL demonstrated similar bacterial growth at 24 hours; an initial concentration of 10^6^ CFU/mL was used across experiments. Samples were incubated for 24 hours following inoculation as both *S*. *pneumoniae* isolates and D39 mutant strains were found to plateau at this time point (data not shown). No difference in CFU/mL of pneumococci was observed in fluids stored for up to 96 hours at 2–8°C following collection.

### *S*. *pneumoniae* proliferated rapidly in all pleural effusions tested

**Fig 1:**
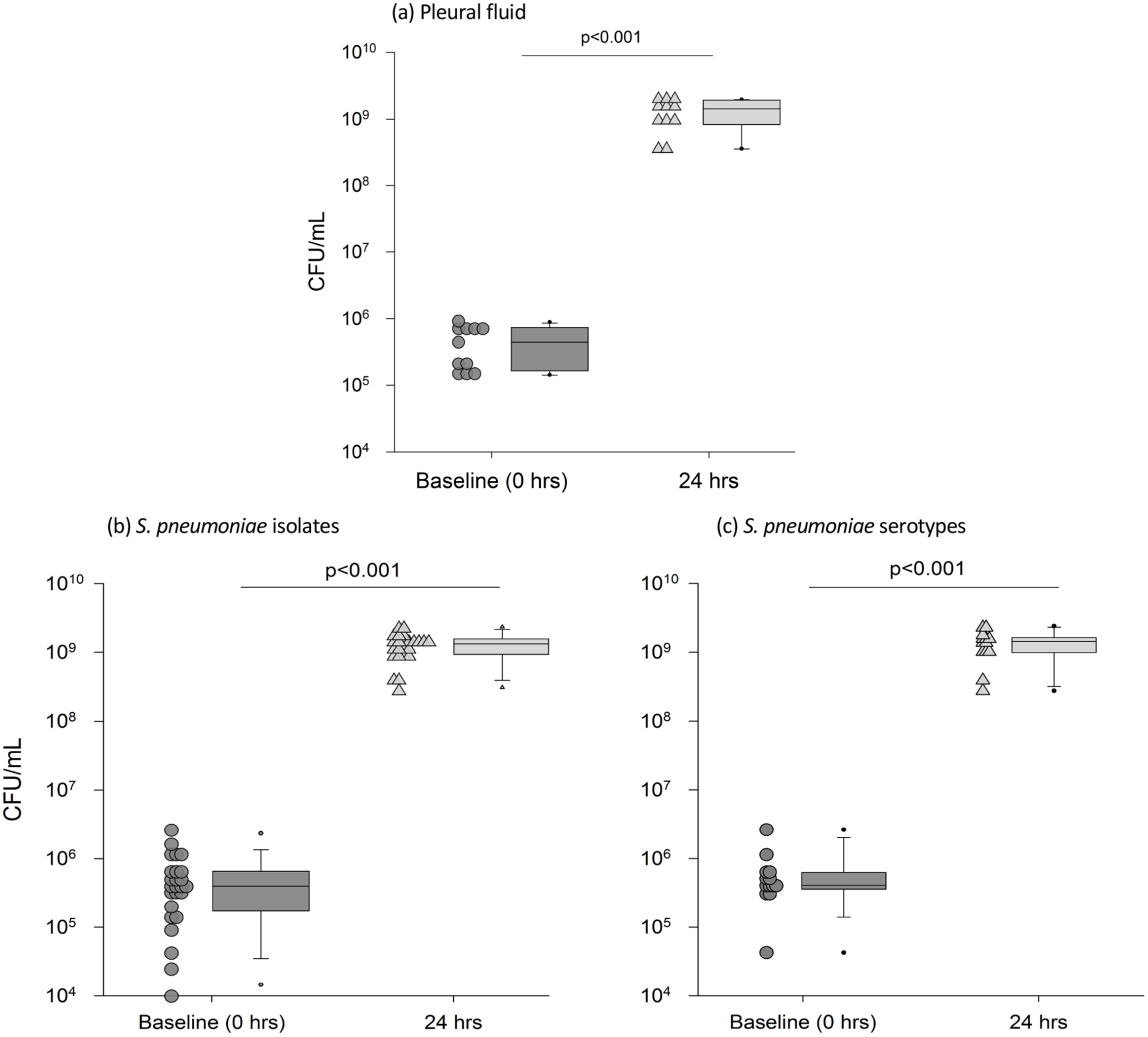
The median growth of *S*. *pneumoniae* (n = 25) in human pleural fluid (n = 11) was consistent across all (a) pleural fluid samples (b) pneumococcal isolates and (c) serotypes. Each dot-point represents data analyzed as (a) the median growth of all *S*. *pneumoniae* in each pleural fluid samples (n = 11), (b) individual *S*. *pneumoniae* isolates (n = 25) and the median growth of each isolate across pleural fluid samples and (c) *S*. *pneumoniae* grouped according to serotype (n = 13); data from the pleural fluid samples (n = 11) is pooled when more than one serotype is available and includes serotypes 1 (n = 2), 6B, 6C, 8 (n = 3), 10A, 11A (n = 2), 12F, 19A (n = 7), 19F, 21, 22F (n = 2), 35B and 3 reference strain. Proliferation was significant at 24hrs across all pneumococci, serotypes and pleural fluids, p<0.001. The box plot represents the median and IQR of the dot plot data; whiskers represent the 95^th^ percentile. Pleural fluid characteristics are presented in Table 3.

All 25 strains of *S*. *pneumoniae* proliferated rapidly in all (n = 11) effusion samples tested (Fig 1) by a median of 3009 fold (IQR 1063–9846) from baseline (from 3.83 × 10^5^ CFU/mL to 1.3 × 10^9^ CFU/mL), p<0.001 after 24 hours. *S*. *pneumoniae* growth was consistent with no difference in CFU/mL when data was analyzed as growth per *S*. *pneumoniae* isolate, growth in each pleural fluid sample or growth per serotype (p = 0.776).

**Fig 2:**
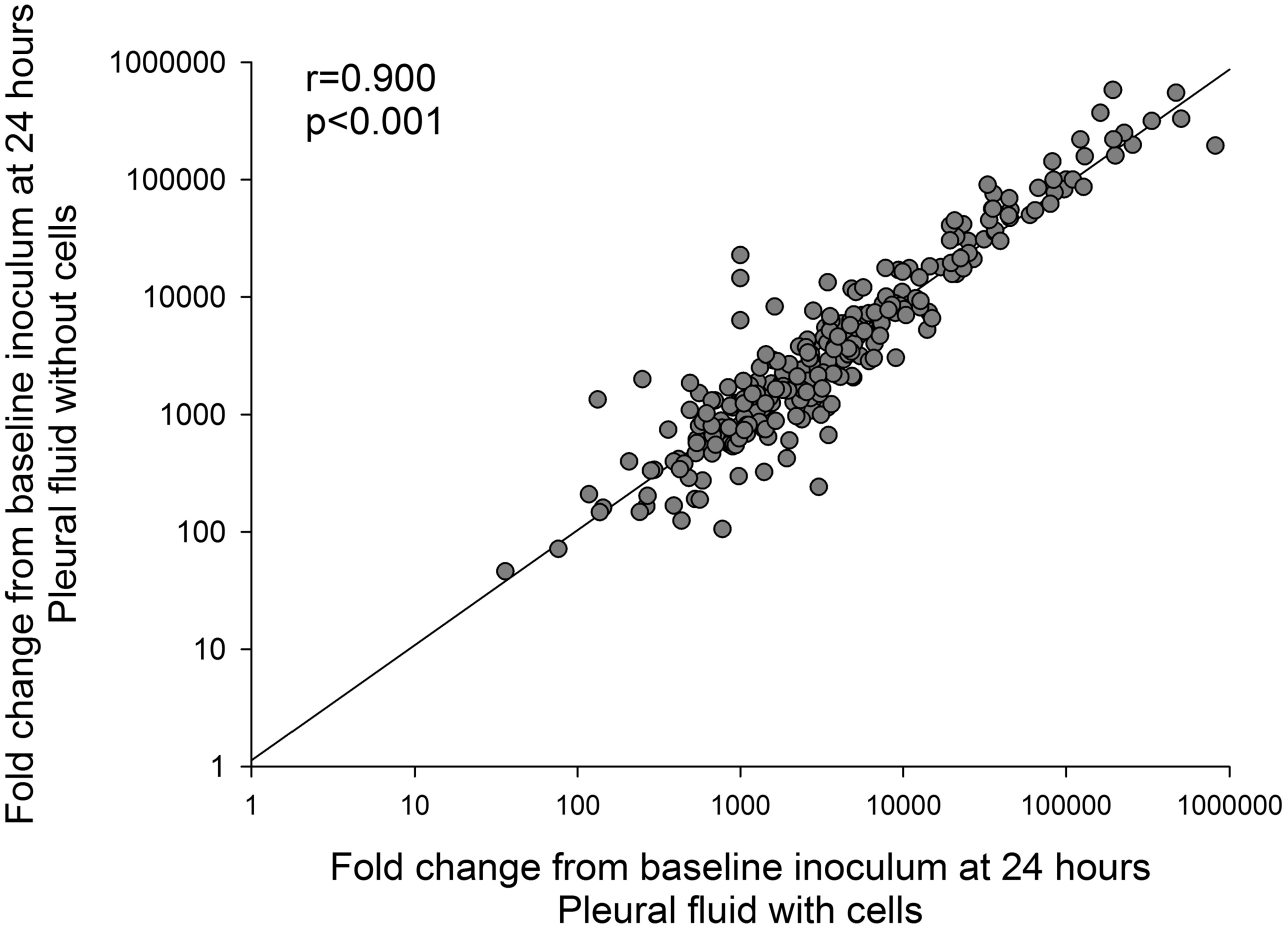
Correlation of *S*. *pneumoniae* (n = 25) growth in pleural fluid with and without cells (n = 11 pairs) expressed as the fold change from baseline inoculum at 24 hours (n = 275 pairs), p<0.001.

Removal of the cellular content of the pleural fluid by centrifugation did not affect *S*. *pneumoniae* growth, with a median fold increase at 24 hours of 2857 (IQR, 1020–9735, p = 0.728 v. growth in uncentrifuged fluid). Growth of *S*. *pneumoniae* in pleural fluid with cells correlated closely to growth in pleural fluid without cells present (Fig 2, p<0.001).

### *S*. *pneumoniae* reaches high densities in pleural fluid and avoids autolysis

**Fig 3:**
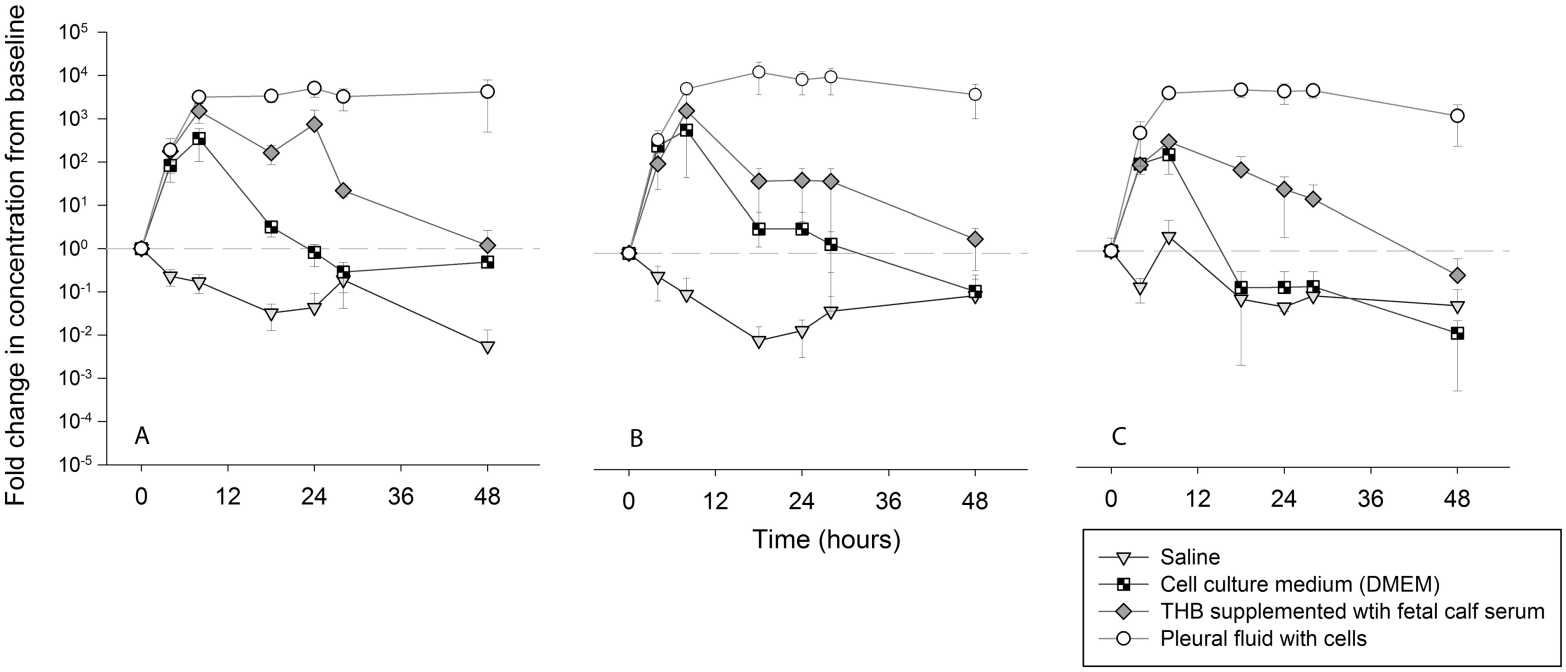
Fold change in CFU/mL from baseline in saline (n = 3), cell-culture medium (DMEM) (n = 3), THB supplemented with fetal calf serum (n = 3) and pleural fluid (n = 6). Each graph represents a different S. *pneumoniae* clinical isolate (a) serotype 8 (blood) (b) serotype 19A (blood) and (c) serotype 19A (pleural fluid). Bacterial CFU/mL in each fluid was determined at 4, 8, 12, 18, 24, 28 and 48 hours. Fluid characteristics are listed in Table 3.

Growth of *S*. *pneumoniae* in pleural fluid was compared to growth in 1) THB with fetal calf serum (17%), a supplemented culture medium commonly used to cultivate pneumococci, 2) DMEM, a glucose rich cell culture medium and 3) 0.85% saline as a control. Rapid proliferation of *S*. *pneumoniae* was observed in THB with FCS, DMEM and pleural fluid within 8 hours following inoculation (Fig 3). The pneumococci did not proliferate in saline alone. Maximum CFU/mL were obtained at 8-hours post-inoculation in broth medium with higher or similar concentrations observed in pleural fluid at the same time point. From 8 to 18 hours’ post—inoculation, autolysis became evident for bacteria grown in either DMEM or culture medium with *S*. *pneumoniae* CFU/mL returning to baseline by 24 to 48 hours. In contrast, concentrations of *S*. *pneumoniae* CFU/mL in pleural fluid were maintained at >1000 fold from baseline over 48 hours.

### *S*. *pneumoniae* strains demonstrated consistent growth whereas growth of other bacterial pathogens was variable

**Fig 4:**
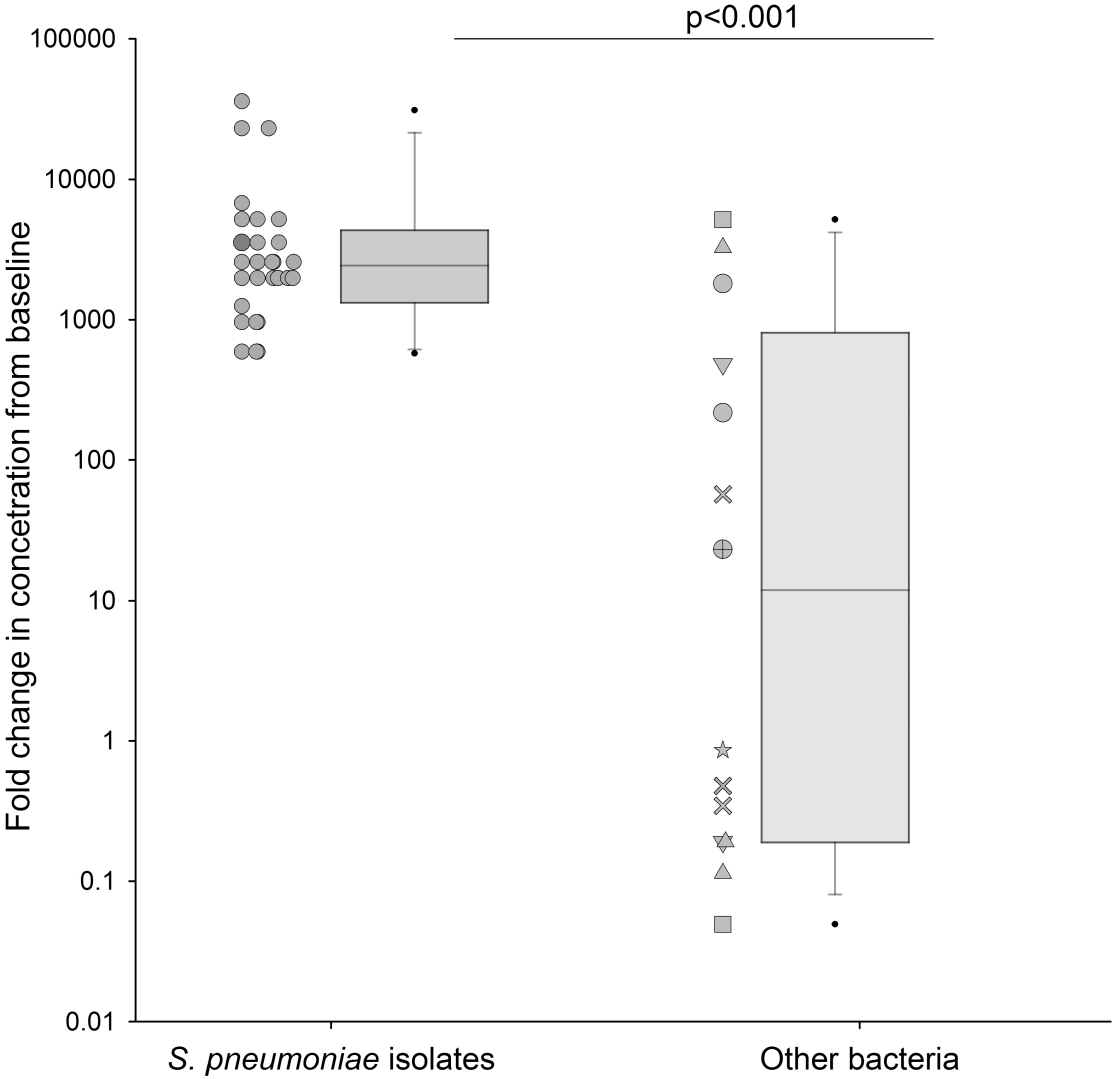
The fold change in CFU/mL of other bacterial pathogens (n = 14) including *P*. *aeruginosa (*△), *S*. *aureus* (О), S. *anginosis* group (×), *E*. *coli* (□), *Klebsiella sp*.(▽), *E*. *faecalis* (⊕), *M*. *catarrhalis* (✯). Each dot point represents the median fold change of each bacterial strain across pleural fluid (n = 7) samples at 24hrs. Data from other bacterial pathogens is compared to previous results of *S*. *pneumoniae* (n = 25) growth in pleural fluid (n = 11), p<0.001. The box plot represents the median and IQR; whiskers represent the 95^th^ percentile.

We investigated the growth of other bacterial pathogens (n = 14) across pleural fluid samples (n = 7). Growth measured at 24 hours was variable across other bacterial pathogens (Fig 4) with a median fold change in CFU/mL of 12.0 (IQR 0.2–813.0) compared to 1172 (IQR, 587–1757) for *S*. *pneumoniae* controls (n = 2), p<0.001. Furthermore, inconsistent growth between strains of the same bacterial pathogen was evident, including within the viridans streptococci group (n = 3) which are a frequent cause of adult empyema. The bacterial pathogens which failed to grow at 24 hours in pleural fluid did not demonstrate increased growth at earlier time points of 4 or 8 hours (data not shown).

### *S*. *pneumoniae* proliferation in pleural fluid is dependent on pneumococcal surface adhesin A (P*saA*)

**Fig 5:**
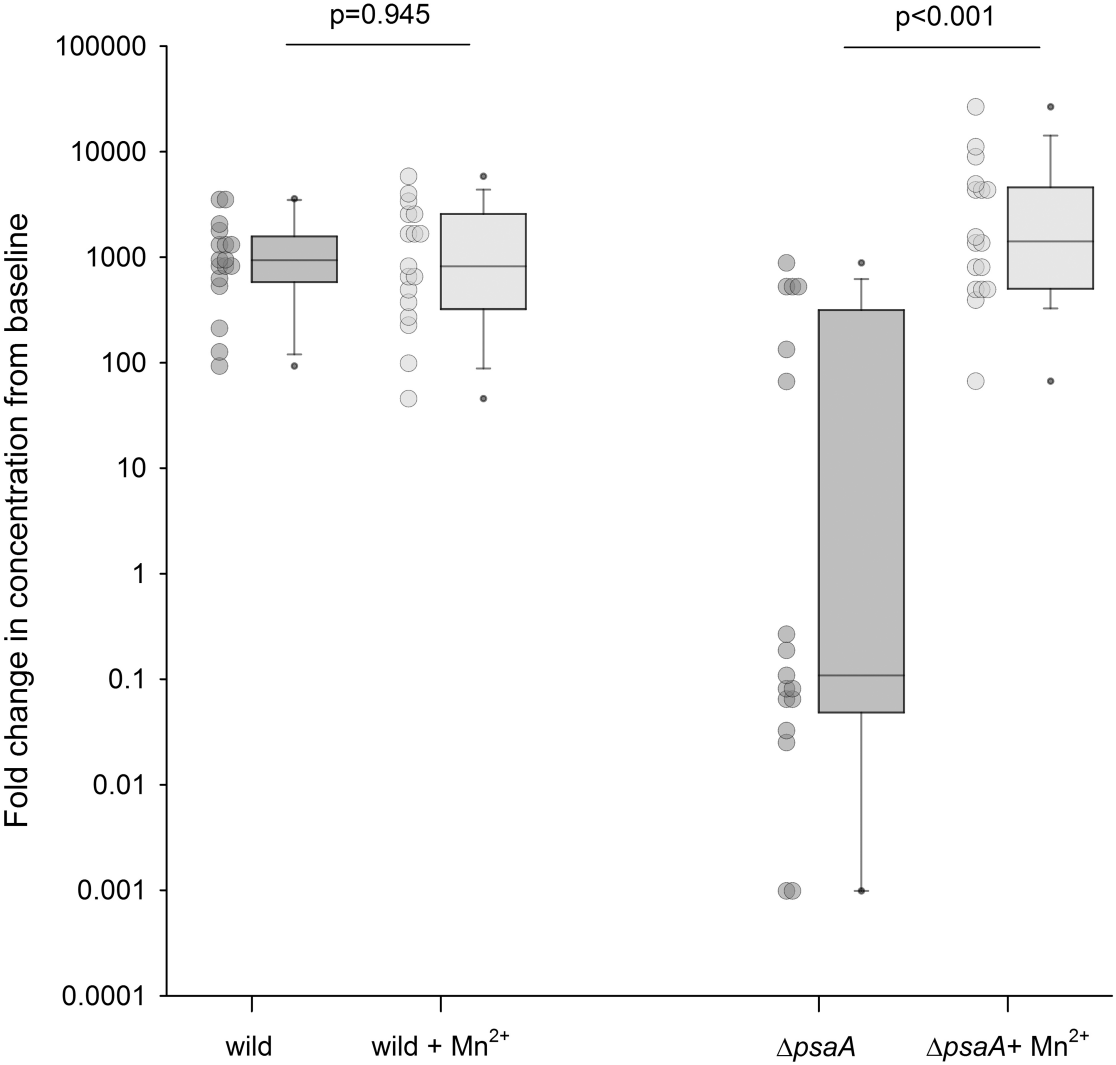
D39 *S*. *pneumoniae* wild type and Δ*psaA* growth in pleural fluid (n = 16) at 24 hrs with and without manganese (Mn^2+^) 3μM supplementation. Δ*psaA* growth was restored with manganese supplementation, p<0.001.

To identify factors required for *S*. *pneumoniae* growth in pleural fluid, growth experiments were repeated using the D39 wild-type strain and eleven mutant strains (*Δply*, *ΔlytA*, *ΔpspA*, *ΔpsaA*, *ΔluxS*, *ΔcbpD*, Δ*pitA*, *ΔpiuB/piaA*, *ΔadcAI*, *ΔadcAII/adcAI*, *ΔlivH*) in eight pleural fluid samples (Table 2). These mutant strains contain mutations in *S*. *pneumoniae* genes required for full virulence either because they encode surface proteins known to be important for host interactions or components of ABC transporters required for nutrient acquisition *in vivo* [11–17, 19, 20, 22]. All of the mutant strains achieved a similar density of CFU/mL as the wild-type D39 stain with the exception of Δ*psaA*. After 24 hours in pleural fluid, the CFU/mL of Δ*psaA* had decreased from baseline with a fold change of 0.55 (IQR, 0.08–3.255) whereas the D39 wild strain had increased 1214-fold (503–1746, p<0.001). A decrease in Δ*psaA* concentrations from baseline was apparent at 4 hours and further decreased at 8 and 24 hours. Importantly, manganese supplementation of pleural fluid by the addition of 3uM of manganese prior to inoculation restored Δ*psaA* proliferation in the pleural fluid, increasing the median fold-change in CFU from 0.11 (0.07–501) to 1546 (509–4775) at 24 hours, p <0.001 (Fig 5). 3μM manganese chloride- tetrahydrate was selected following concentration dependent supplementation experiments ranging from 0.003 to 30μM of manganese. The addition of manganese had no impact on growth of the D39 wild type strain. Iron and zinc supplementation of *Δpit* and *ΔadcAII*/I mutants respectively had no impact on growth compared to the D39 wild type control (data not shown).

## Discussion

*S*. *pneumoniae* is a common cause of both pediatric and adult empyema, yet the reasons why this pathogen can cause pleural infections have only been partially investigated. This is the first study to investigate the interactions of bacteria, namely *S*. *pneumoniae*, and human pleural fluid *ex vivo*. We found that pleural fluid provides a rich medium to support the growth of all relevant *S*. *pneumoniae* strains tested and that pneumococci appear to be much better adapted to grow in pleural fluid than most other common respiratory pathogens. More importantly, growth of *S*. *pneumoniae* in pleural fluid was dependent on PsaA, a manganese transporter making this a potential future therapeutic target. Mutations of other key virulence factors of *S*. *pneumoniae* did not affect the growth of pneumococci in human pleural fluid *ex vivo*.

We have previously shown in our mouse model that *S*. *pneumoniae* from infected pneumonia tissues invades the pleural cavity within 4 hours of infection via transcytosis across the mesothelial cells [23]. Little information exists on the fate of bacteria after they invade human pleural fluid. This is the first study to investigate these interactions.

To investigate the capacity of pleural fluid to support bacterial growth we used human pleural effusions, most with malignant etiology. Parapneumonic effusions and malignant pleural effusions are both exudative in nature and have indistinguishable biochemical compositions with elevated protein and lactate dehydrogenase and reduced glucose and pH. The effusions used for this study display the same biochemical features of non-infective simple parapneumonic effusions. Our results show that all 25 isolates of *S*. *pneumoniae* consistently proliferated across eleven human exudative pleural fluid samples tested with a median increase of approximately 3000-fold from baseline at 24 hours. In contrast, the median growth of other bacterial pathogens in pleural fluid was approximately 10-fold, and interestingly the growth was variable with some bacterial isolates demonstrating similar levels of growth to *S*. *pneumoniae*, whilst other isolates consistently failed to grow.

When tested in parallel, the growth of *S*. *pneumoniae* was similar to optimal laboratory media at 8 hours demonstrating that pleural fluid represents an excellent growth medium for *S*. *pneumoniae*. Furthermore, pneumococci naturally undergo autolysis after reaching stationary phase during growth which was observed in broth at 18 hours, yet bacterial CFU/mL density was maintained for 48 hours after growth in human pleural fluid. This persistent growth is likely to promote significant neutrophil influx and may be a reason why pneumococci are a frequent cause of empyema. This failure of autolysis would ensure *S*. *pneumoniae* strains are capable of maintaining high level infection even in a closed environment such as a pleural effusion. The reasons why autolysis did not occur after growth in pleural fluid are not known. The cellular content of the pleural fluid did not influence *S*. *pneumoniae* proliferation, indicating it is the pleural fluid itself that provides the medium for *S*. *pneumoniae* growth. The ability to grow in pleural fluid did not vary between individual *S*. *pneumoniae* isolates or serotypes investigated.

Multiple cell surface proteins that function as adhesins, in complement resistance, autolytic enzymes and nutrient transporters in *S*. *pneumoniae* have been identified as essential for virulence and growth of the bacterium. We investigated eleven of these virulence factors for their role during growth in pleural fluid, using mutants that are known to have reduced growth in blood (eg *Δply*, *ΔlytA*, *ΔpsaA and ΔlivH)* [11, 20, 24–27] or in cation depleted media *(ΔpiuB/piaA*, *ΔadcAI/ΔadcAII)* [22, 28, 29]. However, perhaps surprisingly, only one mutant strain demonstrated reduced growth in pleural fluid, the Δ*psaA* mutant. PsaA is a lipoprotein required for manganese transport and resistance to oxidative stress [14, 30, 31]. Mutation of PsaA reduces virulence of *S*. *pneumoniae* and growth in laboratory medium that has been depleted of manganese. Providing biochemical complementation of the Δ*psaA* mutation by supplementing pleural fluid with 3uM manganese was sufficient to restore growth in pleural fluid, confirming that it is the low concentration of manganese that causes failure of growth of the Δ*psaA* strain [31]. This suggests that manganese is rate limiting for growth of *S*. *pneumoniae* in pleural fluid yet pneumococci are capable of overcoming this using the PsaA transporter. Potential PsaA inhibitors that have recently been identified open opportunities to control *S*. *pneumoniae* proliferation in the pleural space [32]. Interestingly, growth of *S*. *pneumoniae* with mutations affecting transporters required for iron or zinc uptake was not affected, suggesting that manganese results are specific for growth within pleural fluid.

The study has a number of potential limitations. We were unable to use pleural fluid of infected etiology in the present study for reasons stated in the introduction. However, malignant pleural effusions have a close resemblance in biochemistry to parapneumonic effusions and were therefore used for the majority of our experiments. Although the cellular content of these effusions may differ, we found that the cellular content of the fluid did not influence the growth/proliferation of *S*. *pneumoniae in vitro*. Next, due to the nature of the study, experiments were performed in *ex vivo* pleural fluid and therefore host defenses, including neutrophils that may modify the bacterial proliferation were not accounted for. However, as a proof of principle study our aim was to investigate the direct effect of pleural fluid on *S*. *pneumoniae* proliferation. Furthermore, our data showed no difference in proliferation with or without cellular content of the pleural fluid. Finally, we studied 25 invasive strains of *S*. *pneumoniae* that had caused empyema or septicemia. Future studies that include other serotypes may be needed, though the very consistent nature of the results indicates that the majority of *S*. *pneumoniae* isolates are likely to grow in pleural fluid.

Pleural fluid is a potent growth medium for *S*. *pneumoniae* and may be one reason why pneumococci are a common bacterial cause of empyema. The high density and persistent growth of *S*. *pneumoniae* in pleural fluid highlights the importance of draining infected effusions. Pneumococcal growth was independent of the cellular content of pleural fluid but dependent on the PsaA surface protein, making PsaA a potential future therapeutic target. The variable growth of other bacterial pathogens in pleural fluid may account for the lower incidence of otherwise common respiratory pathogens in empyema and warrants further exploration in order to understand pathogenesis of pleural infection.

## Supporting information

S1 Table Individual pleural fluid characteristics. (DOC)
