# Supplementary material for: Human pleural fluid is a potent growth medium for Streptococcus pneumoniae
Source: PLoS One. 2017 Nov 30;12(11):e0188833. doi: 10.1371/journal.pone.0188833 (PMC5708656; doi:10.1371/journal.pone.0188833)
Supplement: S1 Table — (DOC) [file pone.0188833.s001.doc]

**Data supplement**

**Human Pleural Fluid is a Potent Growth Medium for *Streptococcus pneumoniae***

Natalia D Popowicz ^1,2,3^, Sally M Lansley ^3^, Hui M Cheah ^3^, Ian D Kay ^4^, Christine F Carson ^2^,, Grant W Waterer ^2,5^, James C Paton ^6^, Jeremy S Brown ^7^, Y C Gary Lee ^3,8^*

1. Pharmacy Department, Sir Charles Gairdner Hospital, Perth, Western Australia, Australia
2. Division of Medicine, University of Western Australia, Perth, Western Australia, Australia
3. Institute for Respiratory Health, University of Western Australia Perth, Western Australia, Australia
4. Department of Microbiology and Infectious Diseases, PathWest Laboratory Medicine, Royal Perth Hospital, Perth, Western Australia, Australia
5. Respiratory Department, Royal Perth Hospital, Perth, Western Australia, Australia
6. Research Centre for Infectious Diseases, Department of Molecular and Cellular

Biology, University of Adelaide, Adelaide, South Australia, Australia

1. Centre for Inflammation and Tissue Repair, Respiratory Medicine, University College London, London, England
2. Respiratory Department, Sir Charles Gairdner Hospital, Perth, Western Australia, Australia

*Corresponding author

Email: [Gary.lee@uwa.edu.au](mailto:Gary.lee@uwa.edu.au)

**S1 Table:** Individual pleural fluid characteristics

| Fluid ID | Patient ID | Diagnosis | Age | Sex | pH | LDH | Protein | Glucose |
| --- | --- | --- | --- | --- | --- | --- | --- | --- |
| 1 | 1 | Non-small cell lung cancer | 51 | Female | 7.12 | 4350 | 49 | 0.5 |
| 2 | 2 | Malignant mesothelioma | 70 | Female | 7.45 | 244 | 23 | 5.2 |
| 3 | 3 | Malignant mesothelioma | 58 | Male | 7.31 | 409 | 46 | 3.1 |
| 4 | 4 | Malignant mesothelioma | 57 | Female | 7.43 | 227 | 39 | 6.3 |
| 5 | 5 | Esophageal Cancer | 51 | Female | 7.36 | 116 | 35 | 3.5 |
| 6 | 6 | Malignant mesothelioma | 60 | Male | 7.33 | 480 | 36 | 4.2 |
| 7 | 7 | Malignant mesothelioma | 73 | Male | 7.33 | 303 | 37 | 5.5 |
| 8 | 8 | Malignant mesothelioma | 64 | Male | NA | 267 | 53 | 5 |
| 9 | 9 | Malignant mesothelioma | 75 | Male | 7.15 | 892 | 12 | 0.8 |
| 10 | 10 | Malignant mesothelioma | 59 | Male | 7.41 | 284 | 41 | 4.9 |
| 11 | 11 | Malignant mesothelioma | 75 | Male | 7.39 | 475 | 39 | 6 |
| 12 | 12 | Non-small cell lung cancer | 47 | Female | NA | 234 | 24 | 5.5 |
| 13 | 9 | Malignant mesothelioma | 75 | Male | 7.11 | 463 | 12 | 2 |
| 14 | 13 | Malignant mesothelioma | 80 | Male | 7.3 | 212 | 37 | 3.9 |
| 15 | 14 | Esophageal Cancer | 88 | Female | 7.17 | 597 | 48 | 4.3 |
| 16 | 15 | Bladder Cancer | 70 | Male | 7.35 | 269 | 40 | 6.3 |
| 17 | 16 | Non-small cell lung cancer | 63 | Female | 7.49 | 205 | 23 | 7.6 |
| 18 | 17 | Malignant mesothelioma | 67 | Male | 7.09 | 1400 | 28 | 1.8 |
| 19 | 18 | Malignant mesothelioma | 76 | Female | 7.36 | 252 | 44 | 6.5 |
| 20 | 17 | Malignant mesothelioma | 67 | Male | 7.2 | 2500 | 26 | 1.7 |
| 21 | 18 | Malignant mesothelioma | 76 | Female | 7.37 | 386 | 41 | 7.4 |
| 22 | 19 | Malignant mesothelioma | 80 | Male | 7.33 | 536 | 39 | 3.2 |
| 23 | 20 | Small cell lung cancer | 76 | Female | 7.33 | 214 | 40 | 7.3 |
| 24 | 21 | Ovarian Cancer | 50 | Female | 7.56 | 112 | 26 | 5.6 |
| 25 | 22 | Amyloidosis | 66 | Female | 7.49 | 97 | 10 | 5.8 |
| 26 | 23 | Malignant mesothelioma | 60 | Male | 7.36 | 328 | 30 | 4.5 |
| 27 | 27 | Pancreatic Cancer | 70 | Male | 7.53 | 64 | 12 | 20.2 |
| 28 | 28 | Congestive heart failure | 64 | Male | 7.49 | 76 | 14 | 23 |
| 29 | 29 | Malignant mesothelioma | 74 | Male | 7.18 | 393 | 42 | 2.9 |
| 30 | 30 | Non-small cell lung cancer | 80 | Male | 7.16 | 485 | 34 | 3.7 |
| 31 | 24 | Ovarian Cancer | 76 | Female | 7.29 | 163 | 48 | 3.6 |
| 32 | 25 | Malignant mesothelioma | 66 | Male | 7.23 | 2750 | 20 | 1.4 |
| 33 | 26 | Non-small cell lung cancer | 65 | Male | 7.33 | 280 | 44 | 7.4 |
| 34 | 18 | Malignant mesothelioma | 76 | Female | 7.31 | 322 | 41 | 5.5 |
| 35 | 31 | Non-small cell lung cancer | 73 | Male | 7.21 | 239 | 40 | 3.5 |
| 36 | 23 | Malignant mesothelioma | 60 | Male | 7.27 | 476 | 40 | 4.3 |
| 37 | 32 | Congestive heart failure | 80 | Male | 7.43 | 80 | 13 | 7.6 |
| 38 | 33 | Malignant mesothelioma | 70 | Male | 7.32 | 1230 | 47 | 5 |
| 39 | 34 | Malignant mesothelioma | 71 | Male | 7.32 | 388 | 39 | 4.9 |
| 40 | 35 | Lymphocytosis | 71 | Male | 7.47 | 82 | 17 | 5.5 |
| 41 | 36 | Non-small cell lung cancer | 48 | Female | 7.36 | 567 | 38 | 5.5 |
| 42 | 17 | Malignant mesothelioma | 67 | Male | 7.33 | 596 | 16 | 6 |
| 43 | 37 | Endometrial Cancer | 72 | Female | 7.37 | 1210 | 41 | 4.5 |
| 44 | 23 | Malignant mesothelioma | 60 | Male | 7.29 | 369 | 29 | 3.5 |
| 45 | 38 | Malignant mesothelioma | 66 | Male | 7.36 | 419 | 41 | 5.2 |
| 46 | 39 | Malignant mesothelioma | 75 | Male | 7.06 | 1050 | 30 | 1.6 |
| 47 | 11 | Malignant mesothelioma | 75 | Male | 7.32 | 518 | 36 | 4.6 |
| 48 | 40 | Malignant mesothelioma | 70 | Female | 7.49 | 266 | 18 | 4.9 |
| 49 | 41 | Malignant mesothelioma | 81 | Female | 7.16 | 683 | 30 | 6.4 |
| 50 | 42 | Non-small cell lung cancer | 72 | Female | 7.39 | 245 | 42 | 7.1 |
| 51 | 43 | Congestive heart failure | 76 | Male | 7.3 | 86 | 31 | 3.9 |
| 52 | 44 | Hepatocellular Carcinoma | 71 | Male | 7.52 | 37 | 10 | 10.8 |
| 53 | 45 | Prostate Cancer | 70 | Male | 7.43 | 79 | 33 | 6.4 |

LDH, lactate dehydrogenase
